# Technologies Trend towards 5G Network for Smart Health-Care Using IoT: A Review

**DOI:** 10.3390/s20144047

**Published:** 2020-07-21

**Authors:** Abdul Ahad, Mohammad Tahir, Muhammad Aman Sheikh, Kazi Istiaque Ahmed, Amna Mughees, Abdullah Numani

**Affiliations:** 1 Department of Computing and Information Systems, Sunway University, Selangor 47500, Malaysia; amans@sunway.edu.my (M.A.S.); 19030576@imail.sunway.edu.my (K.I.A.); amna.m@imail.sunway.edu.my (A.M.); 2 Department of Electrical Engineering, COMSATS University, Islamabad 45550, Pakistan; numani.comsian@gmail.com

**Keywords:** smart health-care, 5G, IoT, D2D, cognitive radio

## Abstract

Smart health-care is undergoing rapid transformation from the conventional specialist and hospital-focused style to a distributed patient-focused manner. Several technological developments have encouraged this rapid revolution of health-care vertical. Currently, 4G and other communication standards are used in health-care for smart health-care services and applications. These technologies are crucial for the evolution of future smart health-care services. With the growth in the health-care industry, several applications are expected to produce a massive amount of data in different format and size. Such immense and diverse data needs special treatment concerning the end-to-end delay, bandwidth, latency and other attributes. It is difficult for current communication technologies to fulfil the requirements of highly dynamic and time-sensitive health care applications of the future. Therefore, the 5G networks are being designed and developed to tackle the diverse communication needs of health-care applications in Internet of Things (IoT). 5G assisted smart health-care networks are an amalgamation of IoT devices that require improved network performance and enhanced cellular coverage. Current connectivity solutions for IoT face challenges, such as the support for a massive number of devices, standardisation, energy-efficiency, device density, and security. In this paper, we present a comprehensive review of 5G assisted smart health-care solutions in IoT. We present a structure for smart health-care in 5G by categorizing and classifying existing literature. We also present key requirements for successful deployment of smart health-care systems for certain scenarios in 5G. Finally, we discuss several open issues and research challenges in 5G smart health-care solutions in IoT.

## 1. Introduction

Smart health-care enables advanced diagnostic instruments to provide advanced treatment for the patients and smart health-care devices to enhance the quality of health-care by providing vital signs in real-time. The aim of smart health-care is to facilitate the patients through the delivery of information regarding medical issues and their solutions. Smart health-care enables patients to take appropriate measures in case of critical circumstances [1]. It enables remote check-up service that results in reducing the treatment expenditures and supports health care providers in broadening their services beyond geographical limits. With the expansion of smart cities, a robust smart health-care system is required to guarantee health services to the users. Apart from well-being, one of the significant contributions is reducing health expenditure by timely diagnostics. For instance, Smart health-care with the Internet of Things (IoT) market estimated to be worth 158.1 billion dollars in 2022 [2].

**Figure 1:**
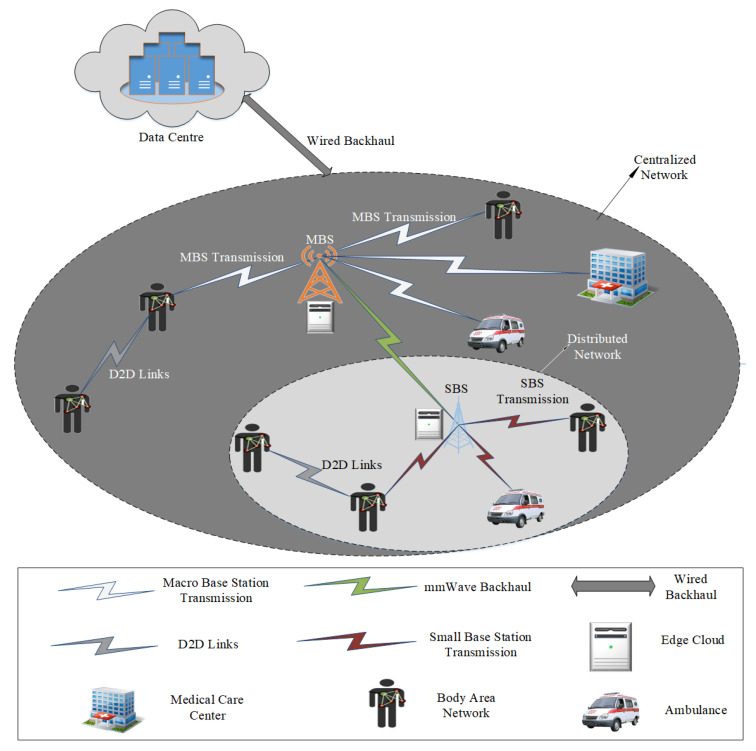
A general architecture of smart health-care network based on 5G.

IoT is going to revolutionize health-care and bring down the cost of medical devices. 5G networks are going to play a major role in order to enable widespread adoption of IoT [3]. In 5G networks, smart health-care is one of the most important applications [4]. Figure 1 shows the general architecture of the 5G-based smart health-care network and its main entities.

In smart-health care, IoT can improve several applications, including asset management in hospitals, behavioural change monitoring, remote monitoring, treatment compliance monitoring, assisted living, smarter medication, and telemedicine [5]. These applications will play an important role in the near future medical business. By 2020, IoT in health care will lead the market to approximately 117 billion US dollars [6]. Many applications for the integration of mobile communication, e-health, and/or web services are proposed. In [7], a portable health application that inspects pressure sore by electronically recording data of health is suggested. In [8], a smart health application for assessment and diet inspection is intended. In [9], the author presents a novel strategy for mobile health applications. In [10], wearable solutions with mobility support are proposed for the living environment. In [11], an IoT application that is based on the mobile gateway is introduced for intelligent assistance in the mobile health environment. In [12], IoT is considered to be an essential factor for medical use in e-health platform. In [13], wearable devices are proposed for inspection of health-care in a wireless network consisting of sensors.

In 5G network communication, smart antennas play an important role [14,15]. Smart Antennas utilize many key innovations to improve 5G coverage and capacity [16,17]. One of the such innovation is beamforming (i.e., vertical and horizontal), in which RF energy is focused in a contract beam to precisely where it is required instead of radiating the same energy in a wide area [18,19]. Beamforming is particularly valuable for 5GNR as the higher frequency mmWave RF is subject to fading over authentication loss and distance caused by objects hitting (e.g., vehicles, buildings, etc.) [20,21]. A more coordinated beam of RF energy helps to guarantee a more prominent probability of ideal transmission capacity and signal quality [22,23]. However, it is imperative to note that line of locate is still an issue, as beamforming points of interest are reduced with attenuation [24,25].

In 5G networks, machine-to-machine (M2M) communication and IoT are expected to be the main pillars of smart health-care [26]. There are two main challenges that the suggested techniques will face. First is a massive number of terminals that have caused ultra-densified networks (e.g., 10^6^ connection per km^2^ approximately. Solutions to address the ultra-densification and scalability problem are needed for IoT and M2M applications. Second is the energy consumption due to the nature of IoT-based applications that are dependent on wireless sensor networks (e.g., the minimum required battery life is 10 years for specific situations [27]). The studies on the deployment and commercialization of 5G network started in 2014 and are expected to completed by 2020 [28]. Besides network densification and support for a massive number of IoT devices, 5G networks are expected to provide a higher data rate. 5G networks are being designed to be flexible and versatile to support new applications, which not only require high data rate, but also other requirements, including massive connectivity, dense deployment, reliability, low-latency, high energy efficiency, and long-range communication to support IoT-based smart health-care applications.

### 1.1. Our Contributions

There have been many studies on smart health-care that explored the topic with various aspects [29,30,31,32,33]. Table 1 shows the review of different researchers related to smart health-care. Our contribution is to deliver a review of 5G smart health-care with a different perspective that includes:

- A taxonomy for smart health-care, covering communications technologies, network types, services, application, requirements, and characteristics.
- Different scenarios for 5G smart health-care and its requirements.
- Key enabling technologies to achieve the requirements of 5G smart health-care and open issues and challenges.

To the best of our knowledge, this is the first paper to present a review of all the above mentioned concisely and comprehensively on the 5G network for smart-health care using IoT.

### 1.2. Organization of This Paper

The remaining paper is arranged, as follows. Section 2 describes a structure for smart health-care, comprising of communication technologies, network types, services, applications, requirements, and characteristics. Section 3 presents scenarios for 5G network and its requirements. Section 4 presents technology trends to achieve the requirements in the 5G network. Section 5 presents open issues and challenges. Finally, Section 6 presents conclusion.

## 2. Taxonomy

**Figure 2:**
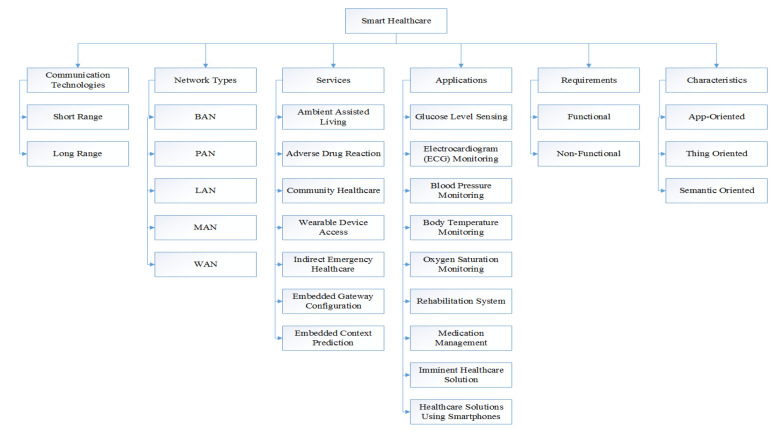
Taxonomy of smart health-care and its parameters.

Figure 2 illustrates the taxonomy of smart health-care. The described taxonomy consists of the following parameters: communication technologies, network types, services, applications, requirements, and characteristics. This section describes the proposed taxonomy in detail in the following sub-section.

### 2.1. Communication Technologies

Various smart health-care services are highly dependent on the communication range (i.e., short and long-range) between devices and servers. Bluetooth, ZigBee, and Wi-Fi are the most noticeable short-range wireless technologies for smart health-care, such as Body Area Network (BAN). LTE and WiMAX are evident long-range technologies used to transfer data between local server to BS in the smart health-care system. Furthermore, LTE-M is considered to be an enhancement to assist IoT. However, 3GPP needs to further provide improvements to address coverage, battery life-time, and device complexity [34]. Besides protocols currently in use, LoRa standardises the LoRa-WAN protocol for smart health-care use to ensure interoperability among several service providers. Moreover, SIGFOX offers an exceedingly adaptable worldwide network by considering smart health-care applications having low power utilisation. A comparison of notable communication technologies is presented in Table 2.

### 2.2. Network Types

IoT-based health-care systems are dependent on various topologies to effectively deliver services. IoT networks provide numerous services on a short-range. For example, Wireless Local Area Networks (WLANs), BANs, and Wireless Personal Area Networks (WPAN) are used in indoor health-care applications. While mobile e-health-care using Metropolitan Area Networks (MANs), Wide Area Networks (WANs), and mobile communication networks. The discussed systems present particular structures in term of size, capacity, data, coverage, and delay requirements.

### 2.3. IoT Health-Care Services

IoT is anticipated to empower a variety of health-care assistance services where every service delivers a combination of health-care solutions. However, health-care and IoT health-care service have no standard definitions. In a few situations, a service cannot be equitably isolated from a specific solution or application. This paper proposes some of the weakness and potential of a service to be a build an application or set of solutions.

Ambient Assisted Living (AAL): AAL are services, concepts, and products that enable technologies and the social environment to enhance the quality of life. The inspiration of AAL is to provide the freedom to elderly people in their place of living in a helpful and safe way. Services that AAL administrations provide self-governance and at the time of any problem providing them remote assistance [35].

Adverse Drug Reaction (ADR): ADR is the reaction happening due to the use of medicine that is prescribed by the doctor. Normally, ADR is the result of using an unusual amount of medicine or perhaps the effect of mixing two or more prescriptions. Although the ADR is inherently generic, which is not particular to the medicine for a specific infection, it is recommended to take few typical specialised problems and their solutions (called ADR administrations). An ADR that is based on IoT is proposed in [36]. In this, the medication is distinguished on the patient’s side through techniques for barcode/NFC-authorized gadgets. With the help of a framework (intelligent in the context of pharmaceutical data), the data are processed out for the detection of medicine to be appropriate with the allergy profile and electronic history of patient’s health.

Community health-care (CH): community health-care services (CHs) are based on the idea of making a local network. This might be a system dependent on IoT throughout the metropolitan health-care centres, rural area, or a residential area. The connection of a few networks like this might be acknowledged as helpful network scenarios. For these scenarios, a specific service, called community health-care (CH), which is unavoidable for meeting aggregate specialised necessities as a suite. A helpful proposal exploiting IoT in rural health-care checking is presented in [37] and observed to be competent in energy consumption. The authorisation and distinct authentication mechanism should be combined because of the cooperative network. In [38], a network for community medication is suggested. This network incorporates numerous Wireless BANs (WBANs) for making CH. The architecture of the network for community medication is just like ’Virtual Medical-Health-care Centre’.

Wearable Device Access (WDA): many types of sensors that are non-intrusive to health have been invented for different medical usage [39], suitable for services of medical health-care dependent on WSN. They are also sufficient for the provision of the same services in IoT. Moreover, wearable gadgets (e.g., smart clothes, smartwatches, and smart glasses) are possible with a group of necessary aspects that are suitable for the design of IoT. However, the demand for different sensors and components in wearable devices is a challenge for the designers and analysts who are working on these integrations, and a service known as WDA can cope with the problem. The unification of wearable gadgets in the WSN-based applications for IoT is explained in [40]. The strategy presents a model that is applicable to a large number of medical health-care applications in different mobile gadgets, such as smartwatches and smartphones.

Indirect Emergency Health-care (IEH): in many situations, health-care services deployments are of crucial importance. These circumstances include incompatible climate conditions, fire accidents, and in-flight etc. In the said situations, a dedicated service known as IEH is capable of arrangements, for example, data accessibility, alter notification, record keeping, and post-accident activity [41].

Embedded Gateway Configuration (EGC): EGC is a compositional facility that is responsible for the connection of nodes (that are specifically associated with patients) in the network, the inter-network (that attaches the essential servers and customers), and more required systems for medical. By a service point of view, the gateway might be emerging with various characteristics, which requires a few common integration features, depending on the particular reason of the conveyed gateway. A good model for an EGC facility is investigated in [42] because of being a component of a universal health-care system. The service permits for computerised along-with intelligent supervising.

Embedded Context Prediction (ECP): for the fabrication of context-aware (CA) medical health-care applications on IoT network, third-party designers require bland systems with appropriate components which are known as ECP service. A similar arrangement is produced in [43] with regards to pervasive health-care. Several challenges in CA health-care system are investigated in [44]. Similar challenges for the mentioned system’s applications when they are overlaid on IoT-based network are thoroughly discussed, a context predictor is used for remote health monitoring that is based on IoT in [45].

### 2.4. Health-Care Applications

Smart health-care services result in the creation of applications, so that clients and patients can correctly use the applications [46]. Table 3 presents different Internet of Things (IoT) based health-care applications.

Sensing of Glucose: diabetes is the gathering of metabolic infections that contain high glucose blood (sugar). The monitoring of blood glucose detect the change in glucose level and help for arranging of diet, physical activities, and time of medication. An m-IoT design method for average glucose instantaneous detection is presented by [47]. The technique that is discussed in this paper is the connection of sensors (associated with patients) by the IPv6 network to the foremost health-care suppliers.

Electrocardiogram (ECG) Supervision: ECG is the inspection of electrical activity record related to human heart, integrates the approximation of the straight-forward pulse and the declaration of vital rhythm along-with the determination of complex arrhythmias, delayed QT intervals, and myocardial ischemia [48].

Blood Pressure Monitoring: blood pressure monitoring detects the signal of pulsation and pressure with the help of sensors, such as electronic pressure and pulsating sensor, and shows the result in digital form. A gadget for collection of blood pressure statistics and sending it over an IoT-based network being presented in [49]. The gadget is composed of blood pressure mechanical assembly with a communication unit. An intelligent terminal in terms of location for continuous blood pressure checking supported by the IoT network is presented in [50].

Body Temperature Supervising: the supervision of body temperature is a fundamental portion of smart health-care. Subsequently, body temperature is a crucial sign in the preservation of stability [33]. In [48], the idea of m-IoT is authorised, utilising a sensor that is monitoring the temperature of the body, implanted in the TelosB, and check of mill trail for achieved temperature readings of the body representing the useful function of the generated m-IoT system is introduced.

Checking Saturation of Oxygen: heartbeat oximetry is appropriate for non-obtrusive observing of oxygen saturation in the blood. The reconciliation of heartbeat oximetry and IoT is valuable for change-driven smart health-care applications. A review of CoAP-based smart health-care discusses the validation of heartbeat oximetry that is based on IoT [51]. Nonin represented the capacity of oximeter for the heartbeat that is wearable on wrist named OX2 in [52]. The device uses sensors that are directly connected to the Monere platform and uses Bluetooth health devices profiles.

System of Rehabilitation: bodily drug and recovery improve and re-establish the useful ability and personal satisfaction of those with physical weakness or disability. The IoT can upgrade the rehabilitation system with the help of medical expert storage. Ontology-based automating strategy for IoT-based rehabilitation system for smart health-care is presented in [53]. This plan effectively exhibits the IoT as a successful step of connecting each essential advantage to offer constant data communications.

Medication Management: generally, health is at risk due to the presence of the resistance issue in medicines and results in a lot of resources wasted throughout the world. When considering the said issue, IoT offers some promising provisions. A bundling technique of pharmaceutical boxes for prescription management based on IoT is presented in [54]. This technique contains a prototypical arrangement of iMedBox and I2Pack and checks the system by field openings. The bundling method comes with precise fixing in need of cleaning constituents controlled by remote correspondences.

Wheelchair Management: many scientists have attempted to create intelligent wheel-chairs assembled with a complete mechanism of debilitated individuals. IoT can help in addressing such issues. A smart health-care scheme for wheel-chair client’s dependent on IoT innovation is presented by [55].

Imminent Health-care Solution: several other convenient health gadgets are available. There is no straightforward mechanism for integrating these gadgets in an IoT system. From time-to-time, these gadgets will be added into IoT systems. Expanding numbers of health software, instruments, and cases are looking to integrate IoT-based health-care throughout the globe. Several health-care services that are looking into integrating IoT are working toward incorporating haemoglobin recognition, top expiratory stream, significant cell genesis, malignancy care, eye problem, contamination of skin, and distant surgery are discussed in literature [56].

Health-care Solutions through Smartphones: in the recent past, the growth of electronic-based gadgets controlled by cell-phone sensors have become prominent and has made cell-phones to be the driver of IoT based smart healthcare solutions. Different hardware and programming objects have been planned to make cell-phones; an adaptable medical services gadget. A detailed study of health-care software for cell-phones is systematically presented in [57]. Additionally, a discussion related to the applications used by patients, general health usage, and medical education is presented in the paper.

### 2.5. Smart Health-Care Requirements

**Figure 3:**
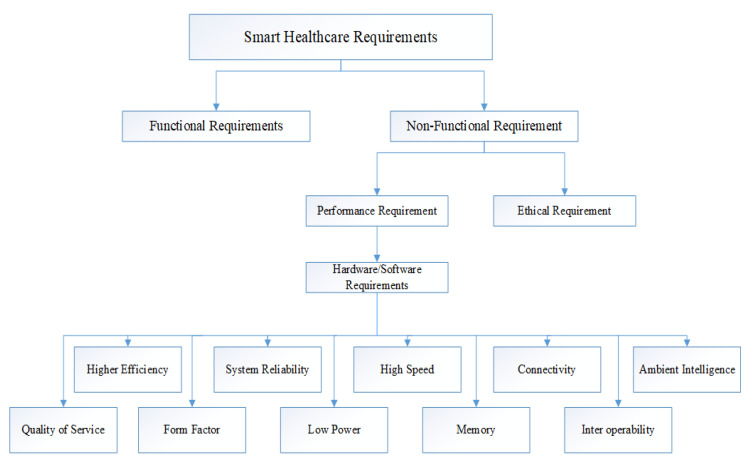
Smart health-care requirements.

Smart health-care requirements may be extensively arranged in functional and non-functional necessities, as presented in Figure 3. Technical specifications deal with the particular needs of the health-care design. For example, the temperature supervising system is organised, in the view of application that it is utilised for, the operation range of thermometer/thermistor, mechanism of data collection, and operation frequency might be different. Therefore, functional requirements are particular to vary segment utilised in that smart health-care system in the light of their application.

On the other side, non-functional specifications are not entirely specific. This requirement implies features through which issues in the health-care framework can be resolved. On a more general point of view, non-functional essentials of health-care may be categorised in ethical and performance specifications. Because of a massive number of barriers being associated with planning an entire intelligent health-care arrangement, performance specifications are additionally characterised in hardware and software specifications. For the smart health-care technique, the basic requirements are system reliability, higher efficiency, small form factor, low power, quality of service, enhanced client encounter, the reputation of the intelligent health-care methodology to provide consistent help, the versatility of method to move up to more up-to-date forms and advances, and abundant availability. Therefore, the specific prime thought process of design and intelligent health-care is to guarantee health service quickly. For cutting edge applications, alongside these specifications, the structure additionally requires ambient intelligence to enhance the nature of the facility.

Points of view of smart health-care broadly differ among researcher and ventures with respect to the picked objective to be accomplished. Parts of intelligent health-care technique may be characterised based on the sensors or actuators, processing devices, information storage components, and network components. A sensor is a sensing device that joins with an organic element that recognises events [58]. Sensors or actuators change, depending on the monitoring system. EMG, blood pressure, ECG, temperature sensors, SpO2, accelerometers, orientation sensors, sensors for motion, and heart rate, are the typical sensors utilised in the smart health-care system. Computing devices used varies from PDAs, smartphone, and tablets to complex and advanced devices, for example, super PCs and servers. Storage assumes an essential job in intelligent health-care, because the storage of data is considered to be the most vital component of the systems. Information repository parts in the creative health-care network ensure a more extensive range beginning from implanted memory on the detecting gadgets to massive servers that are utilised to deal with large information analytics. Network components differ from connected sensors to switches and base stations. In light of the seriousness of the smart-health-care solution tended to, the complexity of the modules changes. Wireless technologies are the foundation of the intelligent health-care network. Distinctive types of wireless technologies, for example, Bluetooth, Wi-Fi, RFID, 6LoWPAN, and so on, as presented in Figure 2, allow for the data to be exchanged among various physical components that are designed to shape the smart health-care network.

### 2.6. Characteristics of Smart Health-Care

**Figure 4:**
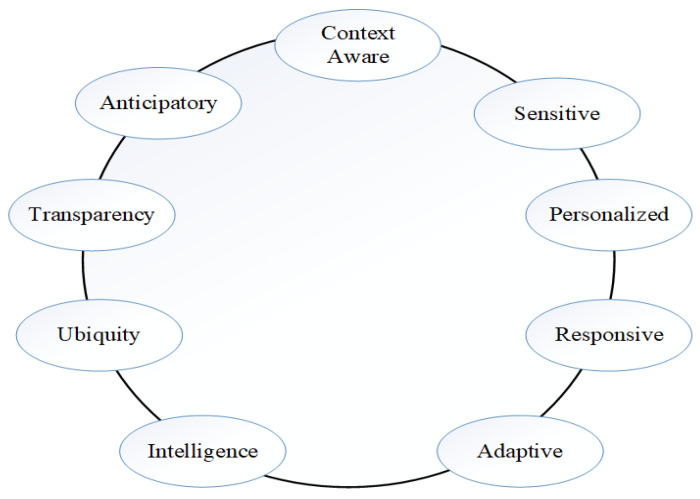
Characteristic of smart health-care.

The critical requirements for successful deployment of the smart health-care system are presented in Figure 4. Those major requirements are classified as Things-oriented, App-oriented, and Semantics-oriented. The establishment of a personalised network in the sensors and the user’s computing device, and information security are the sole responsibilities of App-oriented architectures in order to guarantee the authenticity of data transmissions among applications in smart-phones and sensors. The duties of architectures that are things-oriented are immediate supervision, flexible application, responsiveness at the higher level, consumption of low power, higher efficiency, and switching on the intelligent procedure. Systems that are Semantic-oriented should be capable of handling natural language execution methods for improving user experience, growing detectable specimens that are based on the earlier obtained information, and having exceptional computing abilities [59,60].

## 3. Scenarios for 5G Network and Its Requirements

**Figure 5:**
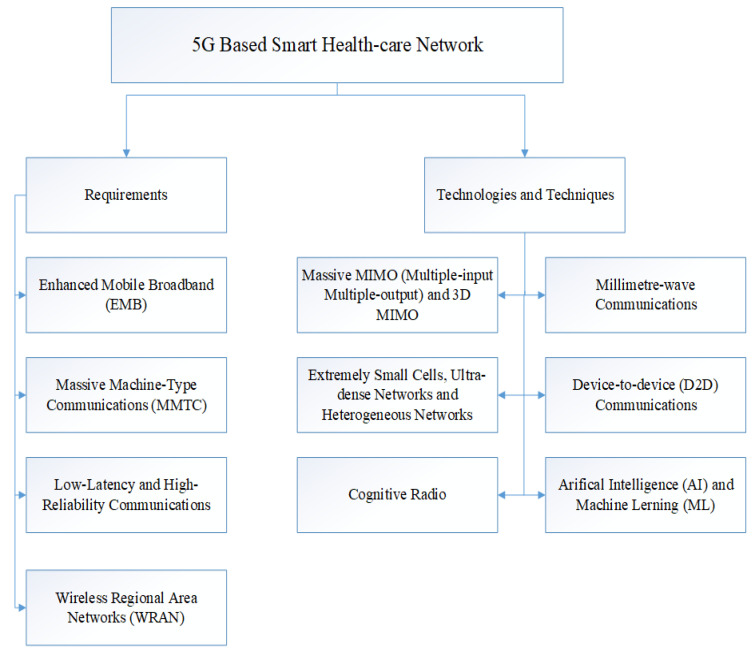
Requirements and technologies trend of 5G based smart health-care.

From the discussion of above Section II, the following four types of scenarios for 5G networks can be classified. Figure 5 illustrates the requirements and the technologies trends to achieve these requirements of 5G based smart health-care network.

- Enhanced mobile broadband.
- Massive machine-type communications.
- High-reliability and low-latency communications.
- WRAN (Wireless Regional Area Networks).

Every setup demands diverse requirements and different applications, discussed as follows.

### 3.1. Enhanced Mobile Broadband (EMB)

The essential requirements of this scenario are high-level throughput and superior traffic capacities. For example, in [61], following requirements are specified; in urban and suburban areas the user-experienced data rate is 100 Mbps and in hotspots, it is up to 1 Gbps; the highest data rate is 20 Gbps; and, the capacity is equivalent to 10 Mbps/m^2^. The main purpose of the scenario is to enhance the traffic capacity and data rate in the network.

### 3.2. Massive Machine-Type Communications (MMTC)

The scenario is related to the wireless sensor network, M2M communication, and IoT communications. The main objective that is related to this scenario is energy efficiency and connection density. For example, in [62], it is stated that the connections density is equal to 10^6^ devices/m^2^ and energy efficiency should be 100 times improved than 4G networks. In [27], it is specified that the devices in the network are required 10-year battery life.

### 3.3. Low-Latency and High-Reliability Communications

The scenario is linked to ultra-reliable communication and Tactile Internet applications, such as remote surgery and vehicle to vehicle (i.e., Ambulances) communications for driverless cars. The main requirement for this scenario is high reliability and low latency. For example, in [63,64], the outage probability that is required is approximately equal to $10^{-7}$ and end-to-end delay is 1ms.

### 3.4. Wireless Regional Area Networks (WRAN)

WRAN is the last setup thought for 5G network. The main objective of this scenario is to provide new application related to remote areas which are lightly populated. The main requirement of this scenario is long-distance communications with cells, which are more significant than 50km in diameter.

## 4. Technology Trends to Achieve the Requirements in the 5G Network

From the above-mentioned applications and scenarios, the essential requirements are summarised in Table 4, which are required for the future generation of the cellular network (5G); ultra-high connection density, traffic capacity and high data rate, ultra-high reliability, ultra-low latency, extremely high energy efficiency, and communication at large distances. For the realisation of these requirements, many technology trends can be identified and how to utilise these technologies to deliver the presented requirements for 5G network. Table 4 show the summary of numerous scenarios for smart health-care and its requirements.

### 4.1. Massive MIMO (Multiple-Input Multiple-Output) and 3D MIMO

According to Shannon’s theorem for the capacity of a network, the upper limit to the capacity of a communication link in bps is fixed, and this rule might be used for investigation on achieving superior data rates [65].

A setup that is working on MIMO, the channel capacity is approximated as

$$C=min\left(M,N\right).Blog_{2}\left(1+SNR\right)$$

**Figure 6:**
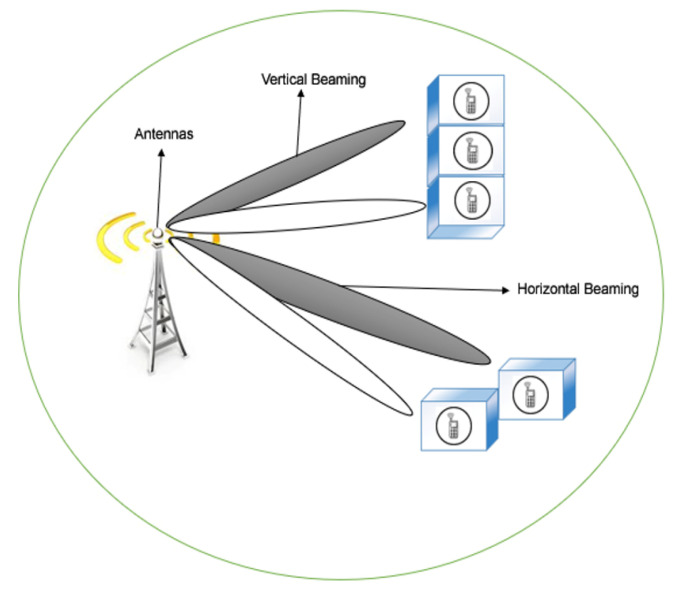
Massive MIMO and 3D MIMO.

*M* and *N* are number of antennas at the transmitter and receiver sides for MIMO system, B is the bandwidth of the channel, while *SNR* is Signal-to-Noise Ratio in the link. From the above theorem, we can obtain that by increasing numbers of antennas in the system, the data rate can be improved. Accordingly, we can use the MIMO system to achieve a high data rate, which can solve the first trend for the next generation network 5G. In massive MIMO, a considerable collection of antennas are utilised in the base station to assist multiple terminals simultaneously [66]. Additionally, by using the active antennas, the control of the antenna beam in both direction horizontal and vertical is possible (called 3D MIMO), this can maximise the cell partitioning [67]. Additionally, SNR can be improved to control antenna beam, which leads to minimising the transmission power or maximising the capacity of the link. Figure 6 shows the idea of MIMO and 3D MIMO. In [68], it was shown that, by using massive MIMO, the capacity can be maximised up to 10 times and the efficiency of radiated energy can be maximised up to 100 times approximately.

### 4.2. Millimetre-Wave Communications

From Equation (1), it is clear that the channel with high bandwidth results in an enhanced data rate. However, for increasing the bandwidth of the channel, the higher operating frequency is required. It will solve the second trend for the next generation network (i.e., 5G) by using higher frequencies in the millimetre wave band. However, there are many disadvantages of millimetre wave communications. Firstly, having free-space propagation in the millimetre wave leads to higher signal attenuation [69]. Secondly, rain, atmospheric gases, and buildings are reasonable observers of this band [70]. For the problem, as mentioned above, different solutions are possible: using tiny cells will lead to minimising the attenuations; for higher attenuations, the directive antennas to be used or indoor base stations to avoid the building absorption issue.

### 4.3. Small Cells, Ultra-Dense Networks, and Heterogeneous Networks

**Figure 7:**
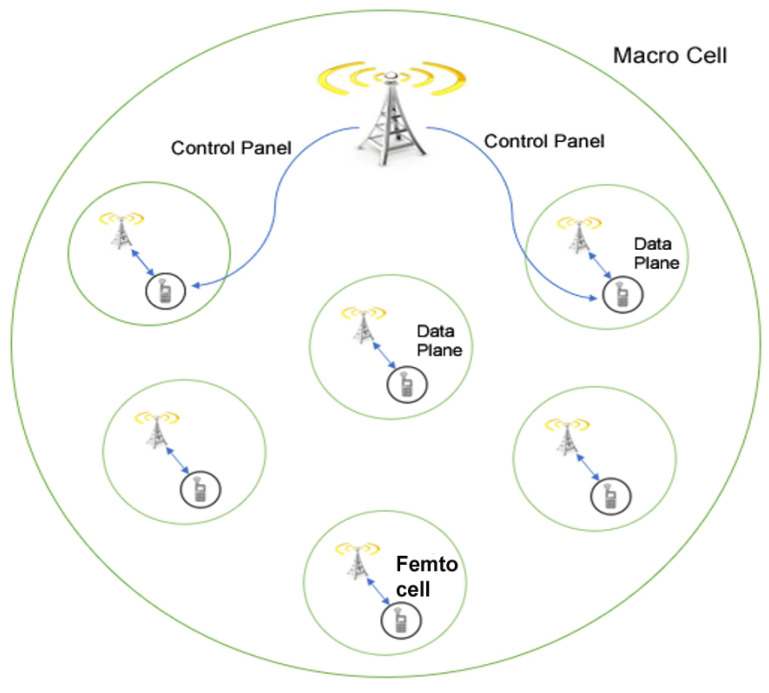
Heterogeneous network.

By the use of small cells, it will enhance the system traffic capacity because of increased frequency re-utilisation. The use of small cells in the scenario of ultra-dense network is an another trend for next-generation network 5G. Furthermore, small cells use also maximises the SNR while the transmission power is minimized; it will reduce the communication power, maximise the link capacity, thereby increasing energy-saving. For 5G, a heterogeneous network with small cells, macrocells, picocell, and femtocell is another trend. In this situation, macrocell support control panel to offer mobility and connectivity, the pico and femtocells support data plane to provide data with transport [66,71,72]. Figure 7 shows this idea. Nevertheless, it is essential to keep in mind that small cells are not suitable in a WRAN scenario. Super microcells can be considered for the WRAN scenario. For this scenario, millimetres wave communication is also not appropriate. Thus, the combination of low frequencies and millimetres wave communications will be considered for the 5G network.

### 4.4. Device-To-Device (D2D) Communications

**Figure 8:**
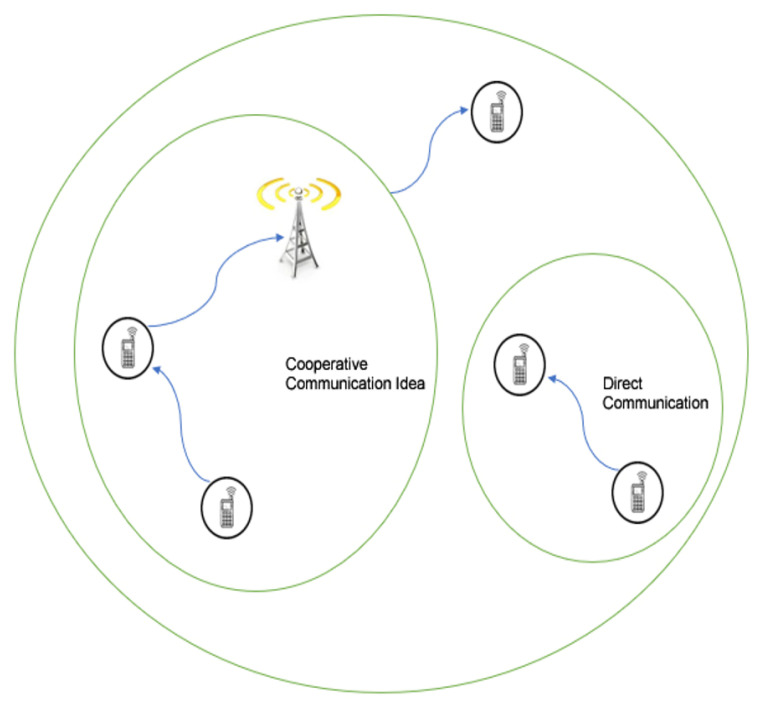
D2D approaches.

D2D communication is another trend for the next-generation network that is 5G [73]. The said technique is usable in two different methods. The first way is where one terminal collaborates with others in a manner that enhances the communication features between the base station and terminals. The second method is where one terminal communicates with another terminal directly without including the base station. Device-to-device communication can improve channel reliability, system throughput, operation cost reduction, and energy efficiency. Figure 8 shows both of the approaches. Direct communication in D2D allows for numerous D2D links concurrently to share the same bandwidth, which leads to increasing the cell traffic capacity. Furthermore, the SNR can be improved with direct communication approach (as compared to the communication through the base station), due to which transmission power is minimised to save energy or maximise the link capacity. At last, the radio link latency is reduced by D2D direct communication [74].

### 4.5. Cognitive Radio

It is another fundamental technology to be considered for the next generation network 5G [75]. Cognitive radio technology might be an essential solution for enabling the WRAN scenario [76]. The users are classified into two categories in the cognitive radio network. The first one is the primary user and the second one is the secondary user. A cognitive system can operate in two modes. The first one is spectrum overlay and the second one is spectrum underlay. During the first approach, secondary users and primary users can transmit simultaneously, while the power of transmission for the secondary user is limited, such that the primary user’s interference is less than a given threshold. In the second approach, the secondary users are allowed to opportunistically utilise the white spaced that are allocated to the primary network in the spectrum using dynamic spectrum access [77]. The Cognitive Network is more appropriated for WRAN scenario.

### 4.6. Artificial Intelligence (AI) and Machine Learning (ML)

Smart health-care applications in the wireless network are still at its early stages and slowly developing with the help of artificial intelligence and machine learning to make the network smarter. The network design, topology, and propagation models, along with the mobility of nodes in the 5G network, can be complicated. Therefore, AI and ML can play vital roles in assisting and managing different resources in the network to make the network smarter for smart health-care applications [78].

AI and ML can be deployed in the network in three different ways.

- For rapid decision making and low computation capability, the AI and ML algorithms can be embedded within individual edge devices in the network.
- For low latency IoT services, AI and ML engines at the network edge can play an important role in performing real-time computation and quick decision making.
- For huge data storage and heavy computation for the analysis of medical data, AI and ML can be embedded in the centralized system to achieve these goals.

## 5. Open Issues and Challenges

Besides the advances that are mentioned above, there are numerous challenges and open research issues in adopting 5G for smart health-care. Table 5 presents the features, advantages, and future research directions. In this section, we describe various research challenges and future research directions.

### 5.1. Achieving Interoperability

Interoperability is a capability to interconnect two or more than two different devices and networks for data exchange. The smart health-care network consists of various IoT devices with different domains (i.e., remote health monitoring, remote surgery, and ECG). Interoperability performs a significant role by providing a platform of connectivity to various devices with different communication technologies. However, due to a lack of universal standards communications technologies, the interoperability between multiple domains is a significant hurdle for IoT success [79]. Therefore, a critical intelligent approach is required to check interoperability at different levels and allow for millions of devices in the network to communicate with each other. Different organisations, like oneM2M and FIWARE, are working in collaboration with various standardisations, like ETSI, OMA, and 3GPP, to sort out the interoperability issue.

### 5.2. Analysis of Big Data

In a smart health-care network, big data analysis is a dominant research direction. The future smart health-care network will consist of millions of devices that will generate an enormous volume of information and data for analysis [80]. These data contain private user information (i.e., Data of Patient) and surrounding environment information of the patient (i.e., Heartbeat rate, ECG, etc.). Therefore, intelligent algorithms and approaches are needed for data analysis. For instance, the information that is generated by local devices in the network must be efficiently analysed with the help of machine learning algorithms. The main concerns that must be addressed are:

- For data analysis privacy must be provided to the user data.
- For sensitive data secrecy must be provided.
- For data collection and analysis, a well-defined infrastructure must be provided.
- For information extraction, computation power must be provided.

### 5.3. Performing IoT Connectivity

The smart health-care network can consist of millions of devices in future. The concept of this network can only succeed if the connectivity is provided to every single device [27]. These devices have the ability to provide information after sensing. In this network, any existing communication technology can be used by IoT devices, such as a cellular network (i.e., 5G and LTE), Bluetooth and Wi-Fi. However, there are many challenges to guaranteeing connectivity to each device in the smart health-care network, such as:

- Guaranteeing connectivity to the devices with high mobility (i.e., moving patient, high-speed ambulance) in the network.
- Providing connectivity to every device deployed in the network with both short and long-range.

### 5.4. Achieving Security, Trust and Privacy

In a smart health-care network, security is an important challenge, due to the connectivity of different IoT devices. It is challenging to implement complex security algorithms and protocols due to limited battery life and processing power on IoT devices [81]. In the future, most of the IoT device will be at risk of attacks. This can lead to different types of threats and attacks in terms of privacy and security. The following issues must be taken into account to design a successful 5G based smart health-care network.

- A secure and straightforward communication must be delivered between smart health-care devices and cloud database centre for data authenticity and integrity.
- Well-defined approach must be provided for risk assessment, to detect upcoming and present attacks.
- Strong privacy policy must be provided for new user approval and trust.

## 6. Conclusions

Next-generation (5G) network are going to play a significant role in smart health-care and IoT applications. Smart health-care and IoT applications have a vital role in the 5G network from functionality and economic point of view. In this paper, we highlighted various applications with diverse perspectives and compared short and long-range communication technologies in terms of range, frequency, power usage, and data rate for smart health-care. Furthermore, four different scenarios are considered according to different requirements for 5G network (i.e., enhanced mobile broadband, low-latency and high-reliability communications, massive machine-type communications, and Internet access for wireless regional area networks), and presented different technology trends for the achievement of these requirements in 5G network and are thoroughly discussed. Finally, we presented the open issues and future research directions related to smart health-care in the 5G network. This provides the opportunities for researchers looking towards starting research within the field of 5G based smart health-care using different approaches, such as machine learning, scheduling, routing, handover, and clustering. There remains an expansive sum of future work due to unique characteristics of smart health-care, and this paper has laid a strong foundation to inspire researchers to investigate the open issues raised in this topic, as mentioned in Table 5.

## Figures and Tables

**Table 1:** Existing survey on smart health-care.

References	Contributions of Authors
Ahad et al. [29]	In this review, the author presented architecture and taxonomy of smart health-care network based on 5G covering the communication technologies, objectives, performance measures, and requirements. Secondly, the author presented a detailed overview of different approaches, such as scheduling and routing, to achieve different objectives and requirements of smart health-care. Finally, the author presented open issues and challenges related to smart health-care.
Mahmoud et al. [30]	In this review, the author presented a review on Cloud of Things (CoT) and how to improved smart health-care applications with the help of CoT. Secondly, the author gave a detailed review of different issues, such as energy efficiency with CoT for smart health-care applications.
Qi et al. [31]	In this review, the author examines different applications of IoT with respect to smart health-care with various aspects (i.e., heartbeat monitoring, oxygen, blood pressure monitoring, oxygen saturation monitoring, etc.). Secondly, the author discussed in detail about existing enable IoT technologies for smart health-care applications with different aspects, such as networking, data processing, and sensing technologies.
Dhanvijay et al. [32]	In this review, the author delivered a detailed review of different IoT smart health-care systems for WBAN, which enables data transmission and data reception. Secondly, the author provided a detailed analysis of security and privacy, power management, resource management, and energy management related to IoT smart health-care.
Baker et al. [33]	In this review, the author proposed a smart health-care model for health monitoring, which can be used for global tracking and special condition monitoring of human being. Secondly, the author delivered a review on the state-of-the-art with respect to different components of the proposed model (i.e., sensors monitoring for blood pressure, wearables that can be monitoring the different condition of the body and vital signs). Thirdly, the author presented a review of different communication standards for smart health-care.

**Table 2:** Comparison of existing proposed wireless communication technologies and their parameters for smart health-care.

	Technology	Types	Frequency	Data Rate	Range	Power Usage
**Short Range Communication**	NFC	PAN	13.56 MHz	100–400 kbps	10cm	Very Low
	Bluetooth 4	PAN	2.4 GHz	1 Mbps	0.1 Km	Low
	Bluetooth 5	PAN	2.4 GHz	2 Mbps	0.25 Km	Very Low
	ISO/IEC 15693	PAN	3.56 MHz	6.6–26 Kbit/s	1–1.5 m	Very Low
	Z Wave	LAN	968–908 MHz	100 kbps	100 m	Very Low
	RFID	LAN	13.56 MHz –2.45 GHz	40–640 kbps	1–100m	Low
	Thread	LAN	2.4 GHz	250 Kbits/s	10–100m	Low
	Wi-Fi	LAN	2. 4 GHz and 5GHz	802.11(b)11 M; (g) 54 M; (n) 0.6, (Gac) 1 Gbps	50 m	Low-High
	ZigBee	LAN	2.4 GHz	250 kbps	10–100 m	Very Low
	WiMAX	WAN	10–66 GHz	11–100 Mbs	50 km	High
**Long Range Communication**	LoRa	WAN	868/915 MHz	50 kbps	25 km	Low
	LoRaWAN	WAN	Numerous	0.3–50 kbps	2–5 km (Urban) 15 km (Sub urban) 45 km (rural)	Low
	Sigfox	WAN	868/915 MHz	300 bps	50 Km	Low
	4G	WAN	700, 1700, 2800 MHz	Up-to 12 Mbps	Up-to 10 Km	High
	5G	WAN	At Low Bands	Up-to 3.6 Gbps	Up-to 10 Km	High
	5G	WAN	At High Bands	10 Gbps	<1 Km	High
	(NB-IoT)	WAN	850 MHz	245 kbps	Up-to 35 Km	High
	(EC-GSM IoT)	WAN	890 MHz	Up-to 140 kbps	Up-to 100 Km	High
	LTE-M (M1)	WAN	700, 1450–2200, 5400 MHz	0.144 Mbps	35km	High

**Table 3:** Internet of Things (IoT) health-care applications.

Infirmity/Condition	Sensor Types	Operations	IoT Role/Connection
Diabetes	Opto-physiological sensor	The output of the sensor is connected with TelosB mote to convert the analogue signals into digital	6LoWPAN, and IPV6 architectures protocol enable all wireless sensors to communicate with wireless nodes that are IP-based
Diabetes Patients injury analysis	Smart-phone camera	Segmentation, and Decompression of image	The application uses smart-phone system-on-chip (SoC) to drive IoT
Monitoring of Heartbeat	Capacitive electrodes on electric circuit	Transmitted information in digital chain, which is connected to the wireless transmitter	Gateway are used to smart devices with the help of Bluetooth and Wi-Fi.
Monitoring of blood pressure	Wearables sensor of blood pressure	Measurement, automatic inflation, and oscillometric.	Smart devices are connected in WBAN with the help of gateway
The temperature of body	Wearables sensor of blood pressure	Measurement of skin-based temperature	Smart devices are connected in WBAN with the help of gateway
System of Rehabilitation	Smart home sensor, full range of wearable sensors.	Tracking, reporting, detection, coordination, cooperation, feedback to the system.	Heterogeneous WSN enable sensors to have many access points.
Management for Medication	Wireless biomedical sensors suit.	Diagnosis and prognosis of essential records. Which are recorded by wearable sensors.	GPS, web access, database access, wireless links, RFIDs and multimedia transmission.
Management of wheelchair	WBAN sensors (ECG, pressure, accelerometers).	Wirelessly communicate with sinks nodes and observe the surrounding.	Data centre layer and smart devices with heterogeneous connections
Monitoring of Oxygen saturation	Pulse oximeter wrist	Intelligent detection of pulse time by time.	Pervasive incorporated clinical environment
Monitoring of skin infection and eye disorder	Smart-phone cameras	Matching of pattern with standard images of the library, visual inspection	The cloud aided application use smart-phone system-on-chip (SoC) to drive IoT
Cough detection	Microphone audio system is installed in a smart-phone	Analysis of recorded spectrograms.	The application uses smart-phone system-on-chip (SoC) to drive IoT
Detection of Melanoma	Smartphone cameras	Matching of the suspicious image with standard images of the library of cancerous skin.	The application uses smartphone system-on-chip (SoC) to drive IoT
Distant surgery	Surgical robot sensors, augmented reality sensors	Robot arms, master controller, and feedback to the user.	Information management and data connectivity in real-time.

**Table 4:** Summary of numerous scenarios for smart health-care.

Scenario	Drivers	Communication Technologies	Required Latency	Required Data Rate
M2M Wearables	Connection for data gathering	NB-IoT (interconnected devices) LoRa (sensor applications) Zigbee (data collection) Bluetooth (D2D sensors)	10–700 ms	Few Kbps to Mbps
Digital Hospital	Communication inside building	Wi-Fi	10–100 ms	Few Mbps
Emergency Medical Services	Emergency Communication and High-speed reply	LTE LTE-A LTE-A Pro	20–100 ms	From 100 Mbps to 3 Gbps
Remote Surgery	URLLC service between many locations	5G	20–30 ms	Few Gbps
Tactile Communication	URLLC (Ultra-reliable and low latency communications), eMBB (enhanced Mobile Broadband)	5G, 4G, Wi-Fi, Bluetooth	sub-ms	Few Gbps
Combination of all scenarios	Communication, latency, bandwidth, applications	5G, 4G, Wi-Fi, Bluetooth	up-to few ms	Few Mbps to 3 Gbps

**Table 5:** Future research challenges.

Features	Advantages	Research Challenges	Key Requirements
Achieving Interoperability	A significant platform for communication between different IoT devices by using various protocols.	Incorporating devices for retailer secured administrations.	Adaptable, universal and integrated models are needed for incorporation and communication (i.e., CoAP, IP) for IoT devices.
Analysis of Big data	Enhance the network performance by processing data received from valid sources (i.e., analysis of patient data with an intelligent method can minimise congestion of network).	Limitation of useful tools to process heavy amount of information generated by devices in the network. Lack of centralized and distributed resources.	Need for a centralized big data centre for processing. Public appreciation on how to utilize available resources with secure manner.
Performing IoT Connectivity	Assurance of the IoT devices communication from various domain.	How to assure the connectivity of various devices from a different domain in high mobility? How to optimize the resources in an ultra-high dense network? How to achieve energy efficiency in an ultra-high dense network?	Usage of the spectrum with an efficient technique for IoT devices communication. Intelligent algorithms that guarantee the connectivity of different devices with various domains in the network. Clustering schemes to support mixed workload and to enhance resource availability.
Achieving Security	Provides a secure platform (free of attacks) to deploy services.	Secure deployment and integration of cloud-based services at both network and device levels. Detection of threats at both levels of insider and outsider before execution. Intelligent security solutions that help in data integrity to prevent delay.	Recognizable proof of vulnerabilities at a different level in the system. which function as entry points for different attacks.

## Abbreviations

The following abbreviations are used in this manuscript:

BAN	Body Area Network
BS	Base Station
CMTC	Critical Machine Type Communication
CoT	Cloud of Things
CDMA	Code Division Multiple Access
CQI	Channel Quality Indicator
D2D	Device-to-Device
EMB	Enhanced Mobile Broadband
ETSI	European Telecommunications Standards Institute
FDMA	Frequency Division Multiple Access
GSM	Global System for Mobile
GPRS	General Packet Radio Service
HetNets	Heterogeneous Networks
IoT	Internet of Things
ITU	International Telecommunication Union
IECR	IoT European Research Cluster
LTE	Long-Term Evaluation
LTE-M	Long-Term Evaluation Advance
LoraWAN	Long Range Wide Area Network
Lora	Long Range
M2M	Machine-to-Machine
mMTC	Massive Machine-Type Communication
MTCs	Machine-Type Communications
MBS	Macro Base Station
MIMO	Multiple-input multiple output
NFV	Network Function Virtualization
NB-IoT	Narrowband Internet of Thing
NFC	Near Field Communication
OFDMA	Orthogonal Frequency Division Multiplexing
OMA	Open Mobile Alliance
QoS	Quality of Service
SNR	Signal-to-Noise Ratio
SBS	Small Base Station
SDN	Software Defined Network
TDMA	Time Division Multiple Access
TCP	Transmission Control Protocol
UHD	Ultra High Definition
UEs	User Equipment’s
URLLC	Ultra-Reliable and Low Latency Communication
WLAN	Wireless Local Area Network
WRAN	Wireless Regional Area Networks
WBAN	Wireless Body Area Network
WiMAX	Worldwide Interoperability for Microwave Access
Wi-Fi	Wireless Fidelity
3GPP	Third Generation Partnership Project
4G	Fourth Generation
5G	Fifth Generation Mobile Network
